# Monitoring drug resistance in *Plasmodium falciparum* clinical isolates collected from Northern Thailand

**DOI:** 10.1186/1475-2875-13-S1-P89

**Published:** 2014-09-22

**Authors:** Chairat Uthaipibull, Patcharaporn Saengratwatchara, Manon Boonbangyang, Parichat Prommana, Sumalee Kamchonwongpaisan, Somdet Srichiratanakool

**Affiliations:** 1 National Center for Genetic Engineering and Biotechnology (BIOTEC), National Science and Technology Development Agency (NSTDA), Pathum Thani, Thailand; 2 Department of Biochemistry, Faculty of Medicine, Chiang Mai University, Chiang Mai, Thailand

## Background

Drug resistance developed by *P. falciparum* is a real threat to malaria disease control and treatment. Recent reports from the Thai-Cambodia and Thai-Myanmar borders have described the artemisinin resistance developed by the parasite. Therefore, there is a need to monitor emergence of resistance parasites in order to limit their spread.

## Materials and methods

Clinical isolates were collected from patients infected with *P. falciparum*. The parasites were adapted in *in vitro* culture and tested for 50% inhibitory concentration (IC_50_) against pyrimethamine (Pyr), chloroquine (CQ), mefloquine (MQ) and dihydroartemisinin (DHA) using standard malaria SYBR Green I-based fluorescence (MSF) assay. Mutations of genes associated with drug resistance were also investigated.

## Results and conclusions

All the tested clinical isolates were resistant to Pyr and CQ, even though Pyr was not used in the area for a long time, while CQ is still used to treat *P. vivax* infection. Only some of the isolates were resistant to MQ, while all of them were sensitive to DHA using standard drug sensitivity assay. Pyr- and CQ-resistant isolates contained mutations at dihydrofolate reductase (*Pfdhfr*) and chloroquine resistance transporter (*Pfcrt*) genes as expected. The results confirm the existence of drug resistance parasites and suggest the use of proper drugs for malaria treatment in the field.

